# “Ancient DNA” reveals that the scientific name for an extinct tortoise from Cape Verde refers to an extant South American species

**DOI:** 10.1038/s41598-021-97064-2

**Published:** 2021-09-02

**Authors:** Christian Kehlmaier, Luis F. López-Jurado, Nayra Hernández-Acosta, Antonio Mateo-Miras, Uwe Fritz

**Affiliations:** 1 Museum of Zoology, Senckenberg Dresden, A. B. Meyer Building, 01109 Dresden, Germany; 2 Departamento de Biología, Facultad de Ciencias del Mar, ULPGC, 35001 Las Palmas de Gran Canaria, Spain; 3 Asociación Paleontológica de Canarias (PALEOCANARIAS), Laboratorio de Paleontología, Facultad Ciencias del Mar, ULPGC, 35001 Las Palmas de Gran Canaria, Spain; 4 Departamento de Zoología, Facultad de Biología de la Universidad de Sevilla, c/ Francisco García González S/N, 41012 Sevilla, Spain

**Keywords:** Herpetology, Palaeontology, Phylogenetics, Taxonomy

## Abstract

We examined the type material of the extinct tortoise species *Geochelone atlantica* López-Jurado, Mateo and García-Márquez, 1998 from Sal Island, Cape Verde, using aDNA approaches and AMS radiocarbon dating. High-quality mitochondrial genomes obtained from the three type specimens support that all type material belongs to the same individual. In phylogenetic analyses using mitochondrial genomes of all species groups and genera of extant and some recently extinct tortoises, the type material clusters within the extant South American red-footed tortoise *Chelonoidis carbonarius* (Spix, 1824). Our radiocarbon date indicates that the tortoise from which the type series of *G. atlantica* originates was still alive during 1962 and 1974. These results provide firm evidence that the type material of *G. atlantica* does not belong to the Quaternary tortoise bones excavated on Sal Island in the 1930s, as originally thought. Thus, the extinct tortoise species remains unstudied and lacks a scientific name, and the name *G. atlantica* has to be relegated into the synonymy of *C. carbonarius*. The circumstances that led to this confusion currently cannot be disentangled.

## Introduction

The rise and global spread of humanity is paralleled by the extinction of many tortoise species^1^. This process seems to have accelerated with the emergence of modern humans, suggesting that non-sustainable harvest played a role for many extinctions in this group of animals matching the paradigm of slow prey^1,2^. Large-bodied and giant tortoises as well as island species were particularly affected^1^. The extinct tortoise fauna of the Cape Verde (Cabo Verde) archipelago could be an example.

**Figure 1:**
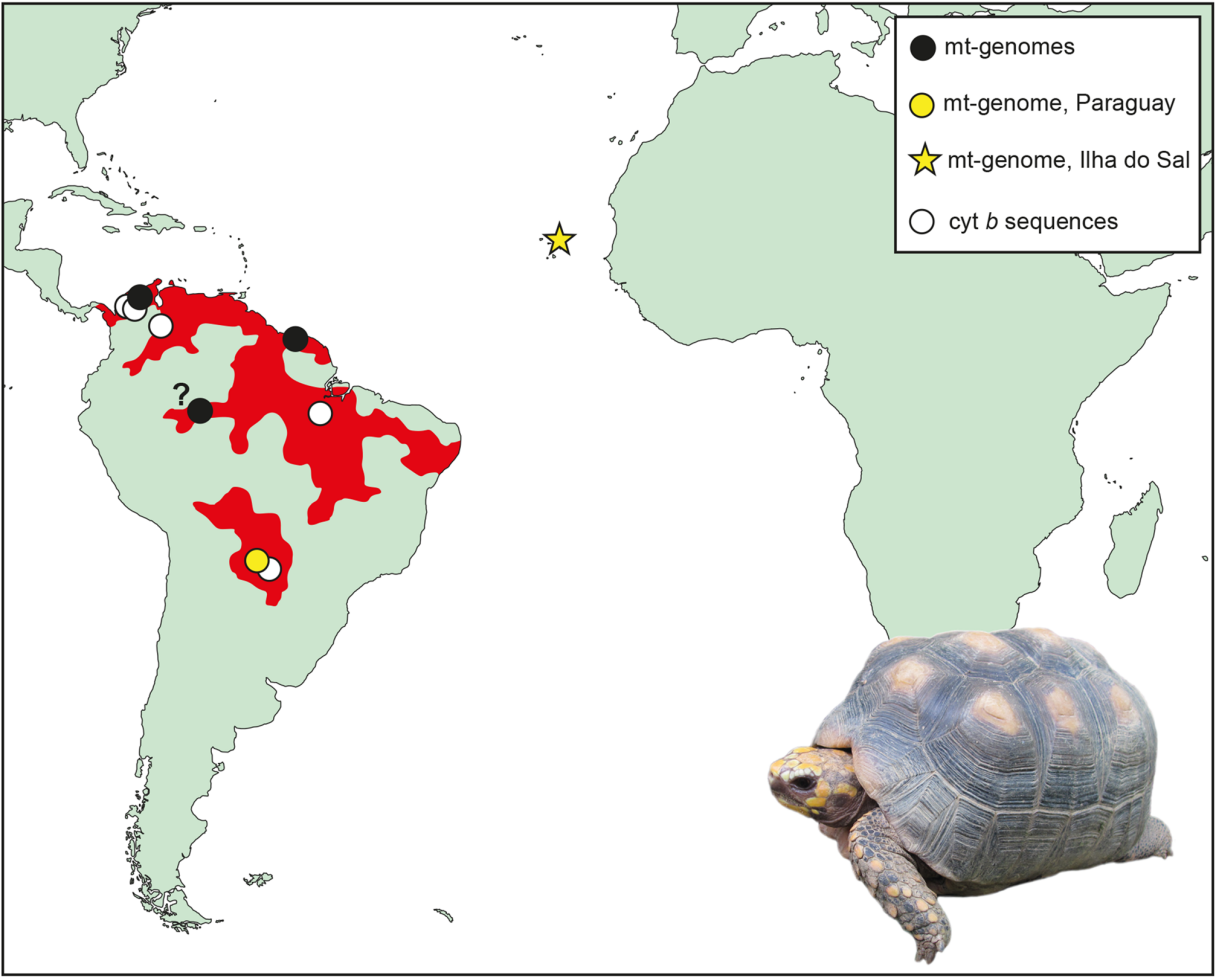
Location of Sal Island (Ilha do Sal), putative collection site of the type material of *Geochelone atlantica* López-Jurado, Mateo and García-Márquez, 1998, distribution range of *Chelonoidis carbonarius*^11^ (red), and collection sites of genetically studied material. The question mark denotes the uncertain collection site of a museum specimen of 1831 (see text). The map was created using ArcGIS 10.4 (https://www.esri.com/en-us/arcgis/about-arcgis/overview) and Adobe Illustrator CS6 (http://www.adobe.com/products/illustrator.html). Inset photo (J. P. Hurtado Gómez): *Chelonoidis carbonarius*.

The Cape Verde archipelago lies approximately 600 km off the Senegalese coast in the central Atlantic Ocean (Fig. 1). It consists of ten small volcanic islands of a combined surface of approximately 4000 km^2^. Biogeographically, the Cape Verdean islands belong with the Azores, Madeira, the Ilhas Selvagens, and the Canary Islands to the Macaronesian region, characterized by many endemics^3,4^. The Cape Verdean archipelago is renowned for its endemic reptiles, in particular for its radiation of endemic skinks that included the recently extinct giant species *Chioninia coctei*^5^. Records of extinct tortoises were described for two Cape Verdean islands. Fossil tortoise eggs are known from the Miocene of Maio Island (Ihla do Maio)^6,7^, whereas younger, “Quaternary,” tortoise bones were described from Sal Island (Ilha do Sal)^7–9^. Sal Island is one of the smaller Cape Verdean islands with a surface of approximately 220 km^2^^10^ and lies in the northeast of the archipelago. It is one of the most arid places on earth. Its Portuguese name Ihla do Sal, island of salt, alludes to its most important natural resource.

Chevalier^8^ and Chevalier et al.^9^ were the first who reported in 1935 tortoise fossils from the Pedra de Lume crater on Sal. This crater of an extinct volcano contains a salt lake from which salt was exploited for centuries, with professional business starting in the eighteenth century. In the nineteenth century, a French company purchased and professionalized the salt production. In the twentieth century up to 40 hectares of saltpans were used for salt production, and a tramway connected the salt refinery to the port. Professional salt production ceased in 1985^10^.

Chevalier et al.^9^ described that they excavated in the Pedra de Lume crater *Phragmites* fossils, providing evidence for the presence of freshwater before the salt lake came into contact with the sea. In another excavation site they found three layers, and assigned all recovered material to the “Quaternary.” In the second site, the uppermost limestone stratum was 150–200 cm heavy. The medium stratum of 40–50 cm consisted of yellowish-white “plates” with plant fossils (“*Sideroxylon marmulana*” = *S. marginatum*), fossil crabs and tortoise bones. The deepest stratum, also limestone, was without fossil remains. According to Chevalier et al.^9^, tortoise bones were very common, both shell fragments and in particular bones from extremities resembling “*Testudo calcarata*,” a large-bodied species from the Sahel region known today as *Centrochelys sulcata*^11,12^.

**Figure 2:**
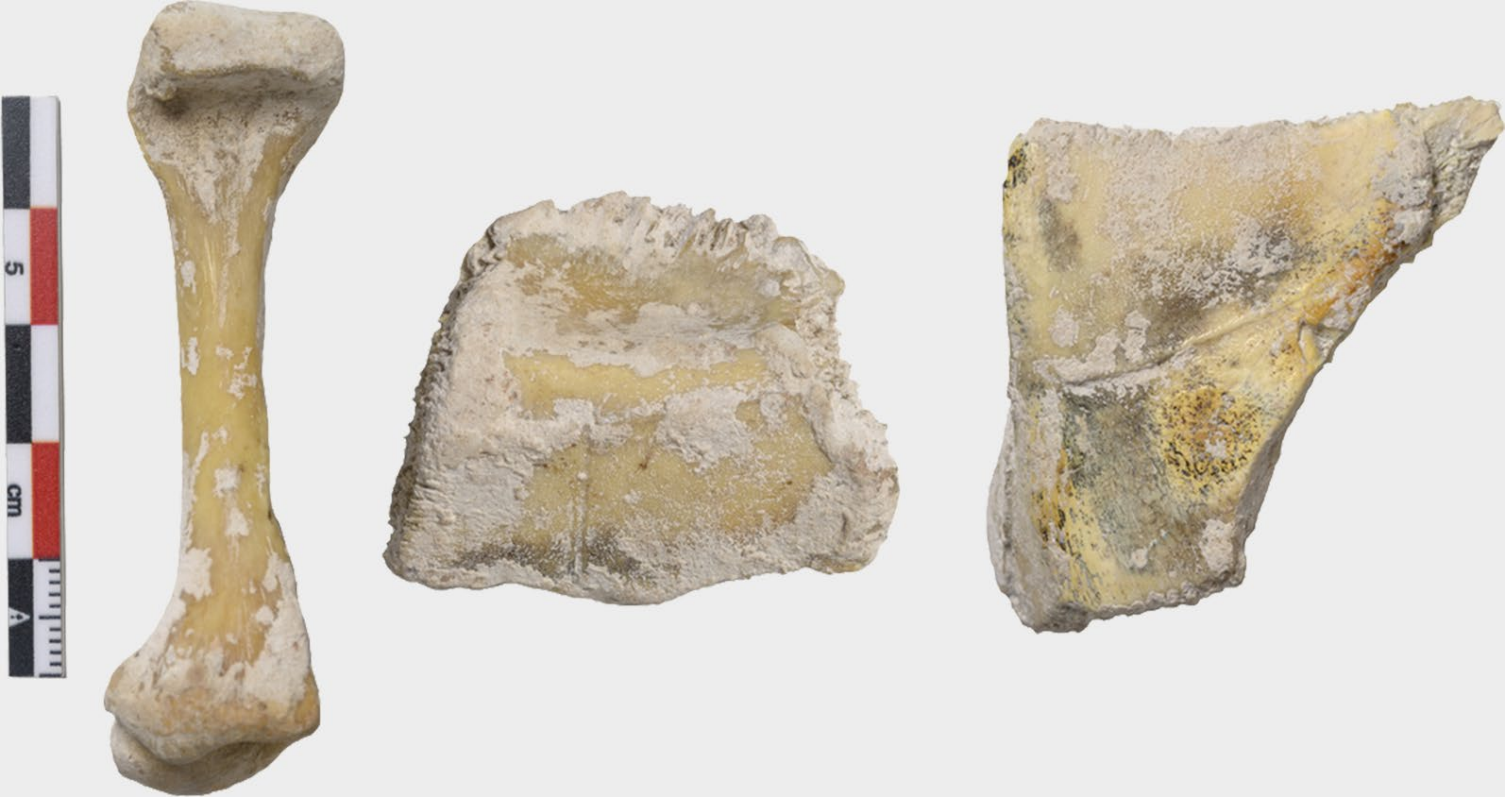
Type material of *Geochelone atlantica* López-Jurado, Mateo and García-Márquez, 1998 in the collection of the Departamento de Biología, Universidad de Las Palmas de Gran Canaria. From the left to the right: holotype (DBULPGC 17, right femur), paratype (DBULPGC 18, second peripheral bone), and paratype (DBULPGC 19, third peripheral bone; photos: N. Hernández-Acosta).

Based on four tortoise bones from the Pedra de Lume crater, López-Jurado et al.^7^ described in 1998 the extinct species *Geochelone atlantica*. These bones were originally in the private collection of Antoine Rivelot (Charenton-le-Pont, France), the nephew of the last French owner of the salt refinery on Sal Island, Jean-Jacques Rivelot. Antoine Rivelot obtained the fossils from his uncle, suggesting that the bones were excavated from the site described by Chevalier et al.^9^. Antoine Rivelot presented a right femur (now the holotype of *G. atlantica*) and two peripheral bones (now paratypes) to the Departamento de Biología, Universidad de Las Palmas de Gran Canaria, Spain (Fig. 2), while a skull embedded in a chalky tufa-like matrix, remained in his private collection. The type material putatively belongs to the same individual^7^.

Despite the comprehensive description of the type specimens^7^, the exact morphology of *G. atlantica* remains unknown due to the fragmentary material. López-Jurado et al.^7^ estimated that the species attained a shell length of approximately 40 cm and suggested that it was closely related to *C. sulcata*, echoing the association with this species by Chevalier et al.^9^. Accordingly, *G. atlantica* was later transferred to the genus *Centrochelys*^1^. However, Georgalis et al.^13^ recently highlighted that no diagnostic characters are known that would justify a reliable generic assignment, which is why they used the genus name in quotation marks to express this uncertainty (“*Centrochelys*” *atlantica*). For simplicity and without taking a stance on its taxonomic relationships, we use in the following the original name combination *Geochelone atlantica* López-Jurado, Mateo and García-Márquez, 1998.

In the present study we examine the identity of the type material of *G. atlantica* using aDNA approaches, continuing our previous investigations on the relationships and biogeography of recently extinct tortoise species^14–16^. We generated near-complete mitochondrial genomes (mt-genomes) for all three type specimens of *G. atlantica* and use these data for placing *G. atlantica* into a phylogenetic framework including representatives of all species groups and genera of extant tortoises plus the extinct giant tortoises from the Bahamas (*Chelonoidis alburyorum*)^14,16^ and the Mascarenes (*Cylindraspis* spp.)^15^. In addition to phylogenetic analyses, we also present an accelerator mass spectrometer (AMS) radiocarbon (^14^C) date for one paratype.

## Results

### Genetics

We succeeded in producing for all three type specimens of *Geochelone atlantica* near-complete mt-genomes of 15,508 and 15,510 bp length in high quality, with a 999- to 2945-fold coverage (Table S1). The three mt-genomes were completely identical, supporting that the holotype and the two paratypes represent the same individual.

**Figure 3:**
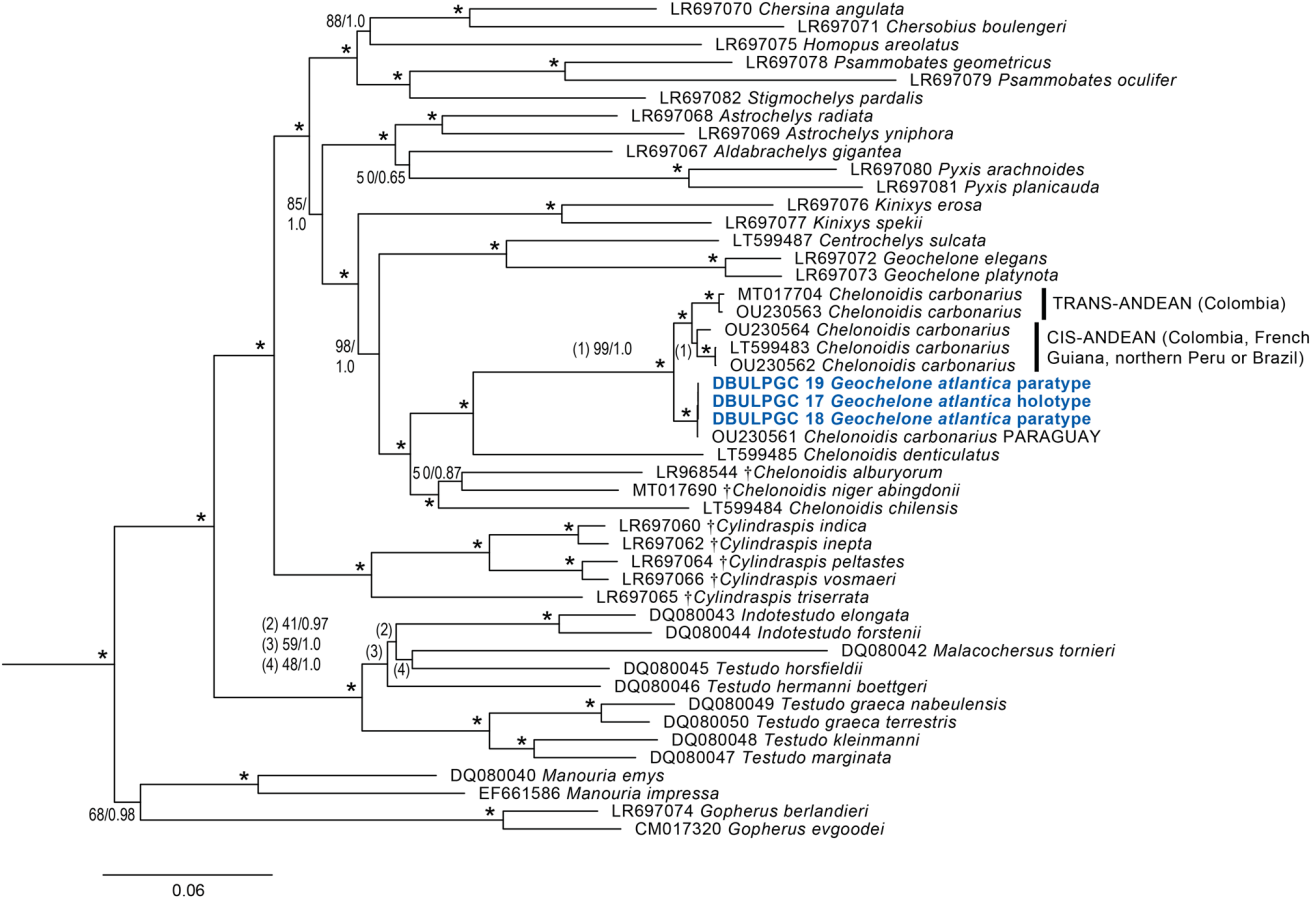
Maximum Likelihood tree for near-complete mitochondrial genomes (15,532 bp) of all species groups and genera of extant tortoises (Testudinidae), some recently extinct taxa and the type material of *Geochelone atlantica* López-Jurado, Mateo and García-Márquez, 1998 (blue). Numbers at nodes are thorough bootstrap values and posterior probabilities from a Bayesian 50% majority rule consensus tree. Asterisks denote maximum support under both approaches. Codes preceding scientific names are ENA/GenBank accession numbers or, for the type material, sample IDs. ENA accession numbers for the type material of *Geochelone atlantica* are OU230558-OU230560. Dagger symbols indicate extinct taxa. Outgroups (*Chrysemys picta*, GenBank accession number AF069423; *Mauremys reevesii*, FJ469674) removed for clarity. Most sequences correspond to a previously published dataset^14–16^ to which four new sequences for *Chelonoidis carbonarius* (OU230561- OU230564) and a new GenBank sequence for *Gopherus evgoodei* (CM017320) were added.

All phylogenetic analyses placed the sequences of the type material in an entirely unexpected position: The South American red-footed tortoise (*Chelonoidis carbonarius*) was paraphyletic with respect to *G. atlantica* (Figs. 3 and S1).

Our mt-genomes of *C. carbonarius* represent all previously identified phylogeographic groups for this species^17^ except for that from northeastern Brazil, for which only previously published sequences of the cytochrome *b* (cyt *b*) gene^18^ were available. Yet, *G. atlantica* clustered both in analyses for the mt-genomes and for cyt *b* sequences with maximum support within *C. carbonarius* and was not differentiated from red-footed tortoises from Paraguay, i.e. from the disjunct southern range portion of *C. carbonarius*. When the mt-genomes were inspected, the sequences of the type material of *G. atlantica* differed only by four mutations from the mt-genome of a Paraguayan red-footed tortoise.

Parenthetically it may be noted that a newly sequenced mt-genome for a historical museum specimen of *C. carbonarius* (MTD D 3620) corresponds to a cyt *b* haplotype from cis-Andean Colombia^17^. The specimen was collected in 1831 either in northern Peru or northern Brazil, suggesting a wide cis-Andean distribution of red-footed tortoises belonging to this cluster.

### Radiocarbon dating of the type material of *Geochelone atlantica*

The studied paratype DBULPGC 19 had a very high pMC value of 143.02 ± 0.32, caused by atmospheric bomb ^14^C due to the nuclear weapon tests in the 1950s and 1960s. The measured value corresponds to the atmospheric value of 1962 and 1974, indicating that the tortoise lived between these two years and probably shortly before or after these dates.

## Discussion

Our study supports that all three type specimens of *Geochelone atlantica* belong to the same individual. More importantly, we provide solid evidence that the type material does not represent an extinct species but the extant South American red-footed tortoise (*Chelonoidis carbonarius*). The type material clusters in phylogenetic analyses using mtDNA with red-footed tortoises from Paraguay, indicating that it originated within the southernmost part of the distribution range of *C. carbonarius*. According to our AMS radiocarbon date, the tortoise to which the type material belongs was still alive between 1962 and 1974.

These results have important implications: (1) The type material of *Geochelone atlantica* López-Jurado, Mateo and García-Márquez, 1998 cannot belong to the bones excavated by Chevalier et al.^9^ in the 1930s; (2) with respect to zoological nomenclature, *Geochelone atlantica* López-Jurado, Mateo and García-Márquez, 1998 is a junior synonym of *Testudo carbonaria* Spix, 1824, now *Chelonoidis carbonarius* (Spix, 1824)^11,12^; (3) the fossil species from the Quaternary of Sal Island remains unstudied and lacks a name; and (4) if the genetically differentiated red-footed tortoises from the disjunct southern distribution range^17^ should be deemed taxonomically distinct in future, the name *Geochelone atlantica* López-Jurado, Mateo and García-Márquez, 1998 has to be used because there exists no older name for these populations^11,12^.

**Figure 4:**
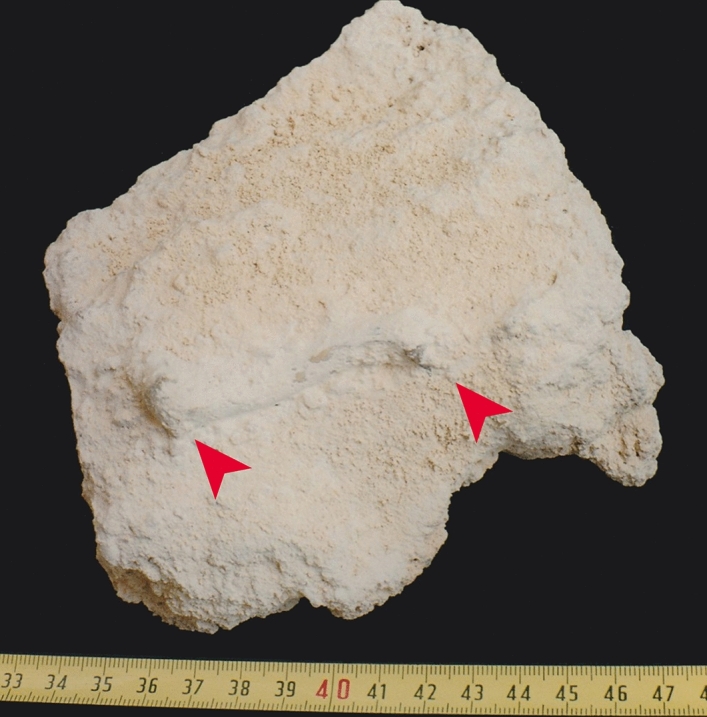
Holotype of *Geochelone atlantica* López-Jurado, Mateo and García-Márquez, 1998 (DBULPGC 17) in original matrix before preparation. Arrowheads point to femoral epiphyses (photo: L. F. López-Jurado).

We can only speculate how bones of the extant red-footed tortoise could have been misidentified as originating from the Quaternary deposits of Sal Island. Before preparation, the type material of *Geochelone atlantica* was embedded in a tufa-like matrix (Fig. 4), as known for Quaternary fossils. One possibility is that the original bones from Sal Island were confused with other superficially similar material, either by the last owner of the salt refinery or in the collection of his nephew, who transferred the type material to the Departamento de Biología, Universidad de Las Palmas de Gran Canaria. Perhaps a *C. carbonarius* was kept as a pet on Sal Island and, after the death of the tortoise, its bones were naturally encrusted in a tufa-like matrix, and later misidentified as belonging to the Chevalier material. Red-footed tortoises were popular pets in Europe during the twentieth century^19^, and somebody living in the French community running the local salt business could have brought the tortoise to the island. However, for the formation of the tufa-like matrix originally covering the type specimens, a humid environment would have been required over several years^20^—quite the opposite of the conditions on the arid Sal Island. Another possibility is therefore that the putative fossils were fabricated and that their matrix has been faked.

Most likely the circumstances that led to this confusion will never be disentangled, even though our present study resolved the taxonomic identity of the type material of *Geochelone atlantica* López-Jurado, Mateo and García-Márquez, 1998. Yet, further research is needed to clarify the whereabouts and the taxonomic identity of the original bone material described by Chevalier et al.^9^.

## Materials and methods

### Genetics

All samples (Table S1) were studied in the molecular genetic laboratories of the Museum of Zoology, Senckenberg Dresden. DNA extraction and library preparation using a bone sample from each type specimen of *Geochelone atlantica* were conducted in a dedicated aDNA facility. It fulfills all requirements for processing aDNA^21^ and is physically isolated from the main laboratory in which fresh material is processed. All subsequent steps after library preparation were performed in the main laboratory. Detailed information on wet lab and bioinformatic procedures can be found in the Supplementary Information (text, Fig. S2, and Tables S2-S7).

For each sample, a near-complete mitochondrial genome (mt-genome) of 15,508 or 15,510 bp length was obtained (Table S1). Each mt-genome covered all 13 protein-coding genes, DNA coding for rRNAs and 21 tRNAs; only tRNA-Pro and the control region were missing.

To unravel the approximate phylogenetic placement of *G. atlantica*, the type sequences were included in a previously published dataset of mt-genomes^15,16^ for all genera and species groups of extant tortoises plus the extinct tortoise taxa from the Bahamas and the Mascarenes and exploratory Maximum Likelihood (ML) analyses were run using MEGA X^22^. *Geochelone atlantica* unexpectedly clustered in these preliminary trees (not shown) with an extant South American species, the red-footed tortoise *Chelonoidis carbonarius*. To examine this situation in more detail, near-complete mt-genomes for one representative each of all mitochondrial haplotype clusters of *C. carbonarius* identified by Vargas-Ramírez et al.^17^ were produced, if samples were still available from the tissue collection of the Museum of Zoology, Senckenberg Dresden (Table S1). In addition, a near-complete mt-genome from a shell of *C. carbonarius* (MTD D 3620) from the herpetological collection of the same museum was generated (see Supplementary Information). This specimen was collected in 1831 by Eduard Poeppig either in northern Peru or northern Brazil. These additional four mt-genomes were added to the final alignment of 15,532 bp that also included a newly released GenBank sequence for *Gopherus evgoodei*.

Using this dataset, phylogenetic relationships were examined using ML and Bayesian Inference (BI) approaches as implemented in RAxML 8.0.0^23^ and MrBayes 3.2.6^24^. The best evolutionary models and partitioning schemes (Supplementary Information) were determined with PartitionFinder2^25^ applying the greedy search scheme and the Bayesian Information Criterion. For ML, 10 independent searches were carried out using the GTR + G substitution model, different starting conditions, and the rapid bootstrap option. Subsequently, 1000 non-parametric thorough bootstrap replicates were calculated and the values plotted against the best tree. For BI, four parallel runs (each with eight chains) were performed with 1 million generations (burn-in 0.25; print frequency 1000; sample frequency 500). Calculation parameters were analysed using Tracer 1.7.1^26^.

In addition, another alignment of 1143 bp length was compiled. It contained all published sequences of *C. carbonarius* for the cytochrome *b* (cyt *b*) gene with a minimum length of 400 bp and homologous sequence data of our new or previously published mt-genomes. Also, one sequence for each other extant or recently extinct *Chelonoidis* species was included. The cyt *b* trees were rooted using *Centrochelys sulcata*, representing a genus that constitutes together with *Geochelone* sensu stricto (*G. elegans*, *G. platynota*) the sister group to all ingroup sequences^15,16^. For this alignment, BI had to be run for 4 million generations before all runs converged as indicated by an average standard deviation of split frequencies below 0.005.

### Radiocarbon dating of the type material of *Geochelone atlantica*

Purified collagen of the paratype DBULPGC 19 (a third peripheral bone from the shell) was radiocarbon-dated in the Leibniz Laboratory for Radiometric Dating and Stable Isotope Research, Christian Albrecht University, Kiel (Germany) using a HVE 3MV Tandetron 4130 system^27^. The obtained conventional ^14^C age was calibrated using the software package OxCal 4.4.2^28^ and the post-bomb atmospheric NH2 data set^29,30^. Further details are presented in the Supplementary Information (Fig. S3).

## Supplementary Information

Supplementary Information.
