# Supplementary material for: “Ancient DNA” reveals that the scientific name for an extinct tortoise from Cape Verde refers to an extant South American species
Source: Sci Rep. 2021 Sep 2;11:17537. doi: 10.1038/s41598-021-97064-2 (PMC8413269; doi:10.1038/s41598-021-97064-2)
Supplement: Supplementary file 1 — Supplementary Information. [file 41598_2021_97064_MOESM1_ESM.pdf]

“Ancient DNA” reveals that the scientific name for an extinct tortoise from Cape Verde refers to an extant South American species

Christian Kehlmaier, Luis F. López-Jurado, Nayra Hernández-Acosta,
Antonio Mateo-Miras & Uwe Fritz

Scientific Reports
https://doi.org/10.1038/s41598-021-97064-2

Supplementary Information

Processing of historical and putatively subfossil samples

The three bone fragments of the type series of *Geochelone atlantica* as well as tissue from inside of a historical shell of *Chelonoidis carbonarius* (MTD D 3620) were analysed in the aDNA facility of the Museum of Zoology, Senckenberg Dresden, which is physically isolated from the main molecular genetic laboratory in another building. Negative controls (water blanks) were included during DNA extraction and library preparation and screened for contamination. The type specimens of *G. atlantica* were sampled using a Proxxon Micromot 50/E multitool equipped with 2–4 mm metal or stone drilling bits. Approximately 50 mg of bone powder of each sample was processed according to a protocol optimised for the recovery of short DNA fragments (Table S1; Dabney *et al*. 2013). According to Gansauge and Meyer (2013) and Korlević *et al*. (2015), 13 ng of obtained DNA was converted into single-indexed, single-stranded Illumina sequencing libraries, including the removal of uracil residues by uracil-DNA glycosylase (UDG) treatment. For *C. carbonarius*, DNA was extracted using Qiagen’s DNeasy Blood & Tissue Kit according to the manufacturer’s protocol. According to Meyer and Kircher (2010), 724 ng of DNA were converted into a single-indexed, double-stranded Illumina sequencing library. In order to increase the amount of endogenous mitochondrial DNA for all four samples, two-rounds of in-solution hybridization capture (Maricic *et al*. 2010; Horn 2012) were performed in a dedicated capture-only workspace in the main laboratory using DNA baits generated from long-range PCR products of *C. chilensis* (for details, see below). Sequencing was performed in-house on an Illumina MiSeq platform, generating 75 bp-long paired-end reads.

Processing of fresh samples

Three blood or tissue samples of *C. carbonarius* previously studied by Vargas-Ramírez *et al.* (2010) and housed in the tissue collection of the Museum of Zoology, Senckenberg Dresden, were examined in the main laboratory. DNA was extracted using commercial blood and tissue kits (Analytik Jena AG). DNA concentration and quality were assessed using a Qubit 3.0 Fluorometer (Thermo Fisher Scientific) and a 4200 TapeStation system (Agilent). For each sample, approximately 15.3 kbp were amplified in two overlapping long-range PCRs (for details, see below). PCR products were sheared to approximately 150 bp with a Covaris M220 ultrasonicator, cleaned with the MinElute PCR Purification Kit (Qiagen), pooled at an equimolar rate, and built into single-indexed double-stranded Illumina sequencing libraries following Meyer and Kircher (2010). Sequencing was as described for the historical material.

Long-range PCR for amplicon sequencing and DNA bait library preparation

For each sample, two long-range PCR reactions were performed (LR1 and LR2), yielding amplicons with an overlap of at least 106 bp and an individual length of approximately 7100–8700 bp, depending on the primer combination (Table S2). For each long-range PCR, a 50 µl volume was used, containing 1 unit of TaKaRa LA *Taq* DNA Polymerase, Hot-Start Version (Clontech Laboratories Inc.) and the reaction mixture recommended by the manufacturer. PCR conditions comprised initial denaturation at 93°C for 3 min, followed by 30 cycles of 93°C for 20 sec, 55°C for 30 sec, 68°C for 10 min, and a final elongation step at 68°C for 20 min. PCR products were visualised and, if necessary, excised from a 2% agarose gel and purified using the NucleoSpin Gel and PCR Clean-up Kit (Macherey-Nagel). The combined long-range PCR products covered most of the mitochondrial genome from the part coding for tRNA-Phe (situated before 12S) to that coding for tRNA-Thr (situated after cyt *b*); DNA coding for tRNA-Pro and the control region were missing.

Authenticity of mtDNA sequenced for the present study

In order to minimize the risk of sequencing nuclear insertions of mtDNA (numts), the following precautions were taken: For fresh samples, long-range PCRs and subsequent amplicon sequencing were performed to minimize the risk of sequencing numts that are typically represented by shorter DNA sequences (Bensasson *et al.* 2001; Fritz *et al.* 2012; Cui *et al.* 2013). During mt-genome assembly, a strict mismatch threshold of 2 was

chosen to prevent the integration of divergent reads of possible nuclear origin. In addition, it was verified that protein-coding DNA sequences contained no internal stop codons.

Bioinformatics

Assembly of mitogenome sequences obtained from single-stranded and double-stranded DNA libraries involved adapter trimming with Skewer 0.2.2 (Jiang *et al*. 2014), read merging (minimum length 35 bp), quality filtering (minimum Q-score 20), and duplicate removal with BBmap-suite 37.24 (https://sourceforge.net/projects/bbmap/; Bushnell *et al.* 2017). The remaining reads were screened for contamination using FastQ Screen 0.11.4 (Wingett and Andrews 2018) and a set of predefined mt-genomes (Table S3). The identified non-target reads were excluded from the readpools. Genome assembly was achieved with MITObim (Hahn *et al*. 2013), a two-step baiting and iterative mapping approach, with an allowed mismatch value of 2. A published sequence of *C. carbonarius* (LT599483) was used as a starting seed. Resulting scaffolds were visualised and checked for assembly artefacts in Tablet (Milne *et al*. 2013). Artefacts were manually removed from the assembled contigs, and all positions with a coverage below 3-fold masked as ambiguous (N) using the maskfasta subcommand of BEDTools 2.29.2 (Quinlan and Hall 2010). Sequence length distribution of mapped reads was calculated with a customised awk command and Microsoft Excel.

The amount of endogenous DNA in the captured aDNA libraries was estimated with MITObim and represents the percentage of mapped reads of the readpool used for genome assembly (Table S1). The software mapDamage 2.0 (Jonsson *et al*. 2013) was used to assess C to T misincorporations, a typical damage pattern of aDNA molecules due to the deamination of cytosine to uracil and subsequent replacement with thymine during the final PCR amplification step of the DNA library preparation. However, due to the young age of the bones and the removal of uracil residues during single-stranded library preparation, the amount of traceable C to T substitutions was negligible.

Preparation of final alignments

The newly generated mt-genomes of *G. atlantica* and *C. carbonarius* were merged with a previously published dataset (Kehlmaier *et al*. 2019, 2021) that contained all species groups and genera of extant tortoises plus some recently extinct taxa from the Bahamas and the Mascarenes. In addition, a newly released GenBank sequence of *Gopherus*

evgoodei (CM017320) was incorporated. In this alignment, 388 bp were removed for calculations (17 bp stop codons, 56 bp gene overlap, 4 bp frameshifts, 311 bp spacer DNA), resulting in a final alignment of 15,532 bp length that comprised 49 sequences. The two outgroup taxa represented the successive sister taxa of Testudinidae, Geoemydidae (*Mauremys reevesii*) and Emydidae (*Chrysemys picta*). A second alignment of 1143 bp length comprised 50 sequences, corresponding to the cyt *b* gene. It included all previously published cyt *b* sequences for *Chelonoidis carbonarius* with a minimum length of 400 bp and one representative of each other *Chelonoidis* species. For this alignment, *Centrochelys sulcata* was used for tree rooting.

Phylogenetic calculations using the two alignments were performed as described in the main text; for further details, see Tables S4–S7.

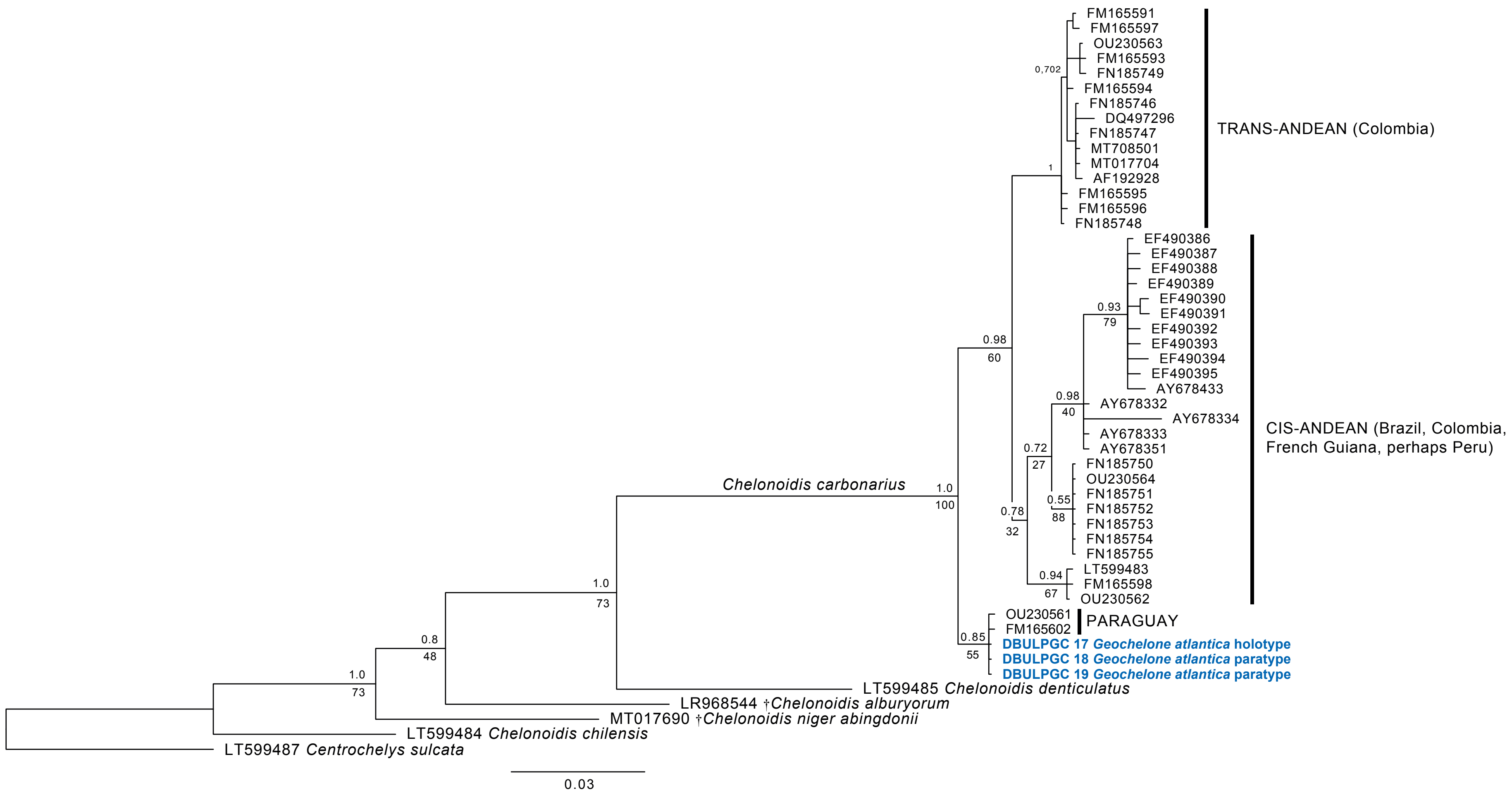

Figure S1. Bayesian 50% majority rule consensus tree for the cyt *b* alignment (1143 bp) including all published sequences for *Chelonoidis carbonarius* ≥ 400 bp and one representative for each other *Chelonoidis* species, rooted with *Centrochelys sulcata*. Sequences for the type material of *Geochelone atlantica* in blue. Numbers at nodes are posterior probabilities and thorough bootstrap values from an ML analysis. Codes preceding scientific names are ENA/GenBank accession numbers or, for the type material, sample IDs. ENA accession numbers for the type material of *Geochelone atlantica* are OU230558–OU230560. Dagger symbols indicate extinct taxa. The clades also contain sequences of unknown geographic provenance.

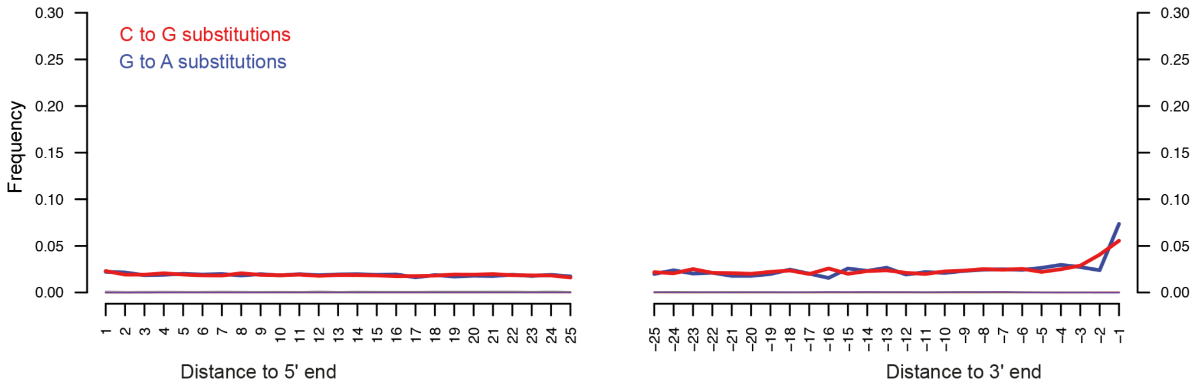

Figure S2. Misincorporation plot generated with mapDamage 2.0 (Jonsson *et al*. 2013) for captured reads of the holotype of *Geochelone atlantica* (DBULPGC 17) mapped to the mitochondrial genome of *Chelonoidis carbonarius* (LT599483). Depicted are the frequencies of substitutions in the reads relative to the reference and their location from the 5' and the 3' read ends.

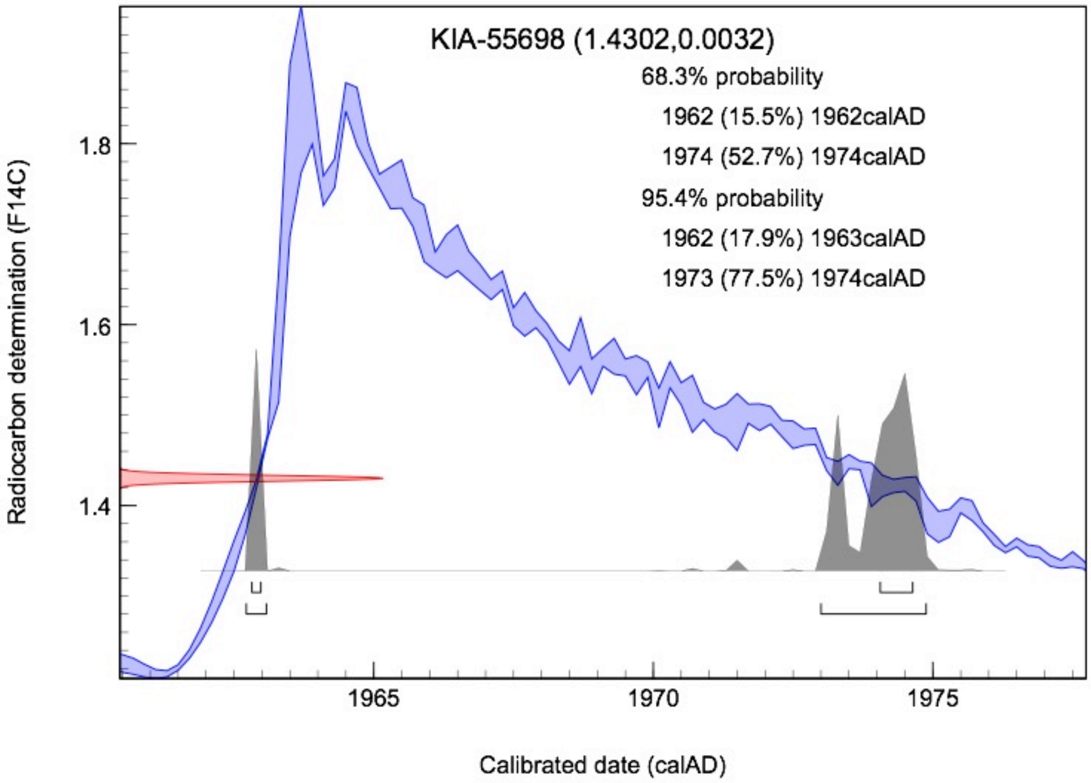

Figure S3. Radiocarbon results for sample KIA-55608 = DBULPGC 19 (paratype of *Geochelone atlantica*) using OxCal 4.4.2 (Ramsey and Lee 2013). The red curve on the left shows the amount of modern carbon including its analytical uncertainty (also given in brackets after the sample code), corresponding to a pMC value of 143.02 ± 0.32. Measurements of known age material (post-bomb atmospheric NH2; Hua *et al*. 2013; Reimer *et al*. 2020) and its uncertainty are shown as the blue double line. The likelihoods of different possible ages of KIA-55608 correspond to the solid grey distributions, providing evidence that the tortoise was alive and incorporated atmospheric radiocarbon between 1962 and 1974.

Table S1. Assembly details of individual samples. Vouchers are in the collection of the Departamento de Biología, Universidad de Las Palmas de Gran Canaria (DBULPGC) and the Senckenberg Natural History Collections Dresden (MTD). Other abbreviations: HT—holotype; PT—paratype; HS—High Sensitivity Assay Kit; ssLib—single-stranded DNA library; dsLib—double-stranded DNA library; ENA—European Nucleotide Archive.

MTD lab no.	Voucher	Taxon	Locality	Type status	Type of DNA library	Dry bone powder into lysis	DNA conc. Qubit HS
—	DBULPGC 17	*Geochelone atlantica*	Cape Verde, Sal, Pedra de Lume	HT	DNA not sheared, ssLib	33 mg	2.1 ng/µl
—	DBULPGC 18	*Geochelone atlantica*	Cape Verde, Sal, Pedra de Lume	PT	DNA not sheared, ssLib	63 mg	2.9 ng/µl
—	DBULPGC 19	*Geochelone atlantica*	Cape Verde, Sal, Pedra de Lume	PT	DNA not sheared, ssLib	57 mg	5.2 ng/µl
8	No voucher	*Chelonoidis carbonarius*	Paraguay, northern Chaco, S20°29 041 W60°18 182	—	amplicon seq, dsLib	n/a	n/a
2087	MTD D 46417	*Chelonoidis carbonarius*	Unknown (pet trade)	—	amplicon seq, dsLib	n/a	n/a
4611	No voucher	*Chelonoidis carbonarius*	Colombia, Departamento del Cesar, Valledupar	—	amplicon seq, dsLib	n/a	n/a
—	MTD D 3620	*Chelonoidis carbonarius*	northern Peru or northern Brazil	—	DNA sheared, dsLib	n/a	36.2 ng/µl

MTD lab no.	Voucher	DNA into library prep	Raw reads	Quality-filtered reads	MITObim assembled reads	Endogenous DNA content	MITObim seed	Average read length in bp	Average coverage	Length of final contig submitted to ENA	Ambiguous sites in final contig	ENA accession number
—	DBULPGC 17	13.0 ng	7,761,499	4,449,249	215,952	4.9%	LT599483	71 bp	999	15,508 bp	31 bp	OU230558
—	DBULPGC 18	13.0 ng	6,479,451	3,593,013	556,631	15.5%	LT599483	80 bp	2945	15,508 bp	5 bp	OU230559
—	DBULPGC 19	13.0 ng	6,039,754	3,882,166	331,781	8.5%	LT599483	82 bp	1780	15,510 bp	12 bp	OU230560
8	No voucher	n/a	692,166	400,246	371,678	n/a	LT599483	n/a	2284	15,359 bp	2 bp	OU230561
2087	MTD D 46417	n/a	637,821	367,117	342,290	n/a	LT599483	n/a	2091	15,362 bp	27 bp	OU230562
4611	No voucher	n/a	664,631	388,384	362,535	n/a	LT599483	n/a	2230	15,360 bp	10 bp	OU230563
—	MTD D 3620	724.0 ng	9,657,355	3,018,233	218,834	7.3%	LT599483	n/a	961	15,486 bp	37 bp	OU230564

Table S2. Primer pairs for long-range PCR.

LR1 For Baits	5´-RTGGCAYTGAAGHTGYCRAGATG-3´
LR1 Rev Chelonoidis	5´-TGGATTATRGCTACTGCTAGTTC-3´
LR2 For Chelonoidis	5´-CTWACAGCYAACCTAACAGCTGG-3´
mt-f-na v2	5´-TCAGTTTTTGGTTTACAAGACC-3´

Table S3. Example of a contamination screening using FastQ Screen for sample DBULPGC 17 (holotype of *Geochelone atlantica*) to assess endogenous DNA content in relation to potential contamination sources. Reported are number of reads that map to a given set of reference mt-genomes. Identified non-target reads, i.e. reads that uniquely mapped to the non-tortoise mt-genomes, were excluded from the final readpool used for subsequent mt-genome assembly.

File	Quality-filtered reads	Unmapped	One hit / one genome	Multiple hits / one genome	One hit / multiple genomes	Multiple hits / multiple genomes
Homo	4,449,249	4,442,726	352	0	6171	0
Penicillium	4,449,249	4,400,466	48,621	143	19	0
Bacillus	4,449,249	4,448,012	42	1020	0	175
Ecoli	4,449,249	4,448,695	87	293	0	174
Felis	4,449,249	4,445,060	2	0	4186	1
Ursus	4,449,249	4,440,471	4	0	8772	2
Canis	4,449,249	4,441,526	6	0	7716	1
Sus	4,449,249	4,441,163	578	0	7505	3
Bos	4,449,249	4,442,246	354	0	6647	2
Gallus	4,449,249	4,438,259	86	0	10,904	0
Mus	4,449,249	4,440,744	65	0	8439	1
Cyprinus	4,449,249	4,441,061	5	0	8183	0
Anaspides	4,449,249	4,447,488	1427	0	334	0
Chelonoidis carbonarius	4,449,249	4,097,133	232,218	21	119,867	10

Table S4. Best evolutionary models and partitioning schemes for the mt-genome alignment as determined by PartitionFinder2 using the greedy search scheme and the Bayesian Information Criterion.

**RAxML**		
**Subset**	**Best Model**	**Partition names**
1	GTR+I+G	Block24 ND6 pos1, Block1 tRNA, Block13 tRNA, Block23 ND5 pos1, Block14 atp8 pos1, Block24 ND6 pos3
2	GTR+I+G	Block4 16S, Block2 12S
3	GTR+I+G	Block26 cyt *b* pos1, Block5 tRNA, Block9 tRNA, Block22 tRNA, Block3 tRNA, Block11 tRNA, Block17 tRNA, Block25 tRNA, Block7 tRNA, Block19 tRNA, Block27 tRNA
4	GTR+I+G	Block18 ND3 pos1, Block20 ND4L pos1, Block6 ND1 pos1, Block8 ND2 pos1, Block15 atp6 pos1, Block14 atp8 pos2, Block21 ND4 pos1
5	GTR+I+G	Block8 ND2 pos2, Block18 ND3 pos2, Block23 ND5 pos2, Block20 ND4L pos2, Block26 cyt *b* pos2, Block15 atp6 pos2, Block6 ND1 pos2, Block21 ND4 pos2, Block12 coxII pos2, Block16 coxIII pos2
6	GTR+I+G	Block21 ND4 pos3, Block15 atp6 pos3, Block14 atp8 pos3, Block23 ND5 pos3, Block20 ND4L pos3, Block16 coxIII pos3, Block24 ND6 pos2, Block8 ND2 pos3, Block6 ND1 pos3, Block26 cyt *b* pos3
7	GTR+I+G	Block10 coxI pos1, Block16 coxIII pos1, Block12 coxII pos1
8	GTR+I+G	Block10 coxI pos2
9	GTR+I+G	Block18 ND3 pos3, Block12 coxII pos3, Block10 coxI pos3
**MrBayes**		
**Subset**	**Best Model**	**Partition names**
1	GTR+I+G	Block18 ND3 pos1, Block20 ND4L pos1, Block14 atp8 pos2, Block6 ND1 pos1, Block8 ND2 pos1, Block21 ND4 pos1, Block15 atp6 pos1, Block27 tRNA, Block14 atp8 pos1, Block24 ND6 pos3, Block1 tRNA, Block23 ND5 pos1
2	GTR+I+G	Block2 12S, Block4 16S
3	GTR+I+G	Block3 tRNA, Block25 tRNA, Block22 tRNA, Block19 tRNA, Block7 tRNA, Block5 tRNA, Block26 cyt *b* pos1, Block9 tRNA, Block11 tRNA, Block17 tRNA
4	GTR+I+G	Block6 ND1 pos2, Block26 cyt *b* pos2, Block15 atp6 pos2, Block21 ND4 pos2, Block16 coxIII pos2, Block12 coxII pos2
5	GTR+I+G	Block8 ND2 pos3, Block6 ND1 pos3, Block26 cyt *b* pos3, Block14 atp8 pos3, Block24 ND6 pos2, Block16 coxIII pos3, Block21 ND4 pos3, Block23 ND5 pos3, Block15 atp6 pos3, Block20 ND4L pos3
6	HKY+I+G	Block8 ND2 pos2, Block18 ND3 pos2, Block23 ND5 pos2, Block20 ND4L pos2
7	SYM+I+G	Block12 coxII pos1, Block16 coxIII pos1, Block10 coxI pos1
8	HKY+I	Block10 coxI pos2
9	GTR+I+G	Block18 ND3 pos3, Block10 coxI pos3, Block12 coxII pos3
10	HKY+I+G	Block24 ND6 pos1, Block13 tRNA

Table S5. Data blocks of the mt-genome alignment used for phylogenetic analyses.

Block1 tRNA	= 1-27;
Block2 12S	= 28-1043;
Block3 tRNA	= 1044-1118;
Block4 16S	= 1119-2809;
Block5 tRNA	= 2810-2886;
Block6 ND1 pos1	= 2887-3864\3;
Block6 ND1 pos2	= 2888-3864\3;
Block6 ND1 pos3	= 2889-3864\3;
Block7 tRNA	= 3865-4078;
Block8 ND2 pos1	= 4079-5116\3;
Block8 ND2 pos2	= 4080-5116\3;
Block8 ND2 pos3	= 4081-5116\3;
Block9 tRNA	= 5117-5498;
Block10 coxI pos1	= 5499-7040\3;
Block10 coxI pos2	= 5500-7040\3;
Block10 coxI pos3	= 5501-7040\3;
Block11 tRNA	= 7041-7175;
Block12 coxII pos1	= 7176-7862\3;
Block12 coxII pos2	= 7177-7862\3;
Block12 coxII pos3	= 7178-7862\3;
Block13 tRNA	= 7863-7941;
Block14 atp8 pos1	= 7942-8112\3;
Block14 atp8 pos2	= 7943-8112\3;
Block14 atp8 pos3	= 7944-8112\3;
Block15 atp6 pos1	= 8113-8781\3;
Block15 atp6 pos2	= 8114-8781\3;
Block15 atp6 pos3	= 8115-8781\3;
Block16 coxIII pos1	= 8782-9561\3;

Block16 coxIII pos2	= 8783-9561\3;
Block16 coxIII pos3	= 8784-9561\3;
Block17 tRNA	= 9562-9631;
Block18 ND3 pos1	= 9632-9979\3;
Block18 ND3 pos2	= 9633-9979\3;
Block18 ND3 pos3	= 9634-9979\3;
Block19 tRNA	= 9980-10052;
Block20 ND4L pos1	= 10053-10343\3;
Block20 ND4L pos2	= 10054-10343\3;
Block20 ND4L pos3	= 10055-10343\3;
Block21 ND4 pos1	= 10344-11717\3;
Block21 ND4 pos2	= 10345-11717\3;
Block21 ND4 pos3	= 10346-11717\3;
Block22 tRNA	= 11718-11933;
Block23 ND5 pos1	= 11934-13754\3;
Block23 ND5 pos2	= 11935-13754\3;
Block23 ND5 pos3	= 11936-13754\3;
Block24 ND6 pos3	= 13755-14289\3;
Block24 ND6 pos2	= 13756-14289\3;
Block24 ND6 pos1	= 13757-14289\3;
Block25 tRNA	= 14290-14359;
Block26 cyt *b* pos1	= 14360-15502\3;
Block26 cyt *b* pos2	= 14361-15502\3;
Block26 cyt *b* pos3	= 14362-15502\3;
Block27 tRNA	= 15503-15532;

Table S6. Best evolutionary models and partitioning schemes for the cyt *b* alignment as determined by PartitionFinder2 using the greedy search scheme and the Bayesian Information Criterion.

**RAxML**		
**Subset**	**Best Model**	**Partition names**
1	GTR+G	Block1 cyt *b* pos1
2	GTR+G	Block1 cyt *b* pos2
3	GTR+G	Block1 cyt *b* pos3
**MrBayes**		
**Subset**	**Best Model**	**Partition names**
1	HKY+I	Block1 cyt *b* pos1
2	HKY+I	Block1 cyt *b* pos2
3	HKY+G	Block1 cyt *b* pos3

Table S7. Data blocks of the cyt *b* alignment used for phylogenetic analyses.

Block1 cyt *b* pos1	= 1-1143\3;
Block1 cyt *b* pos2	= 2-1143\3;
Block1 cyt *b* pos3	= 3-1143\3;

References

Bensasson, D., Zhang, D.-X., Hartl, D. L. & Hewitt, G. M. Mitochondrial pseudogenes: Evolution's misplaced witnesses. *Trends Ecol. Evol.* **16**, 314–321 (2001).

Bushnell, B., Rood, J. & Singer, E. BBMerge—accurate paired shotgun read merging via overlap. *PLoS One* **12**, e0185056 (2017).

Cui, H. *et al*. Comprehensive next-generation sequence analyses of the entire mitochondrial genome reveal new insights into the molecular diagnosis of mitochondrial DNA disorders. *Genet. Med*. **15**, 338–394 (2013).

Dabney, J. *et al*. Complete mitochondrial genome sequence of a Middle Pleistocene cave bear reconstructed from ultrashort DNA fragments. *Proc. Natl. Acad. Sci. USA* **110**, 15758–15763 (2013).

Fritz, U. *et al*. Molecular phylogeny of Central and South American slider turtles: Implications for biogeography and systematics (Testudines: Emydidae: *Trachemys*). *J. Zool. Syst. Evol. Res.* **50**, 125–136 (2012).

Gansauge, M.-T. & Meyer, M. Single-stranded DNA library preparation for the sequencing of ancient or damaged DNA. *Nat. Protoc.* **8**, 737–748 (2013).

Hahn, C., Bachmann, L. & Chevreux, B. Reconstructing mitochondrial genomes directly from genomic next-generation sequencing reads—a baiting and iterative mapping approach. *Nucleic Acids Res.* **41**, 1–9 (2013).

Horn, S. Target enrichment via DNA hybridization capture. In *Ancient DNA: Methods and Protocols* (eds Shapiro, B. & Hofreiter, M.), 177–188 (Springer, Berlin, 2012).

Hua, Q., Barbetti, M. & Rakowski, A. Z. Atmospheric radiocarbon for the period 1950-2010. *Radiocarbon* **55**, 2059–2072 (2013).

Jiang, H., Lei, R., Ding, S. W. & Zhu, S. Skewer: A fast and accurate adapter trimmer for next-generation sequencing paired-end reads. *BMC Bioinform.* **15**, 182 (2014).

Jonsson, H., Ginolhac, A., Schubert, M., Johnson, P. L. F. & Orlando, L. MapDamage2.0: Fast approximate Bayesian estimates of ancient DNA damage parameters. *Bioinformatics* **29**, 1682–1684 (2013).

Kehlmaier, C. *et al.* Ancient mitogenomics clarifies radiation of extinct Mascarene giant tortoises (*Cylindraspis* spp.). *Sci. Rep.* **9**, 17487 (2019).

Kehlmaier, C. *et al.* Ancient mitogenomics elucidates diversity of extinct West Indian tortoises. *Sci. Rep.* **11**, 3224 (2021).

Korlević, P. *et al.* Reducing microbial and human contamination in DNA extractions from ancient bones and teeth. *Biotechniques* **58**, 87–93 (2015).

Maricic, T., Whitten, M. & Pääbo, S. Multiplexed DNA sequence capture of mitochondrial genomes using PCR products. *PLoS One* **5**, e14004 (2010).

Meyer, M. & Kircher, M. Illumina sequencing library preparation for highly multiplexed target capture and sequencing. *Cold Spring Harb. Protoc.* **2010**, pdb.prot5448 (2010).

Milne, I. *et al.* Using Tablet for visual exploration of second-generation sequencing data. *Brief. Bioinform.* **14**, 193–202 (2013).

Quinlan, A. R. & Hall, I. M. BEDTools: A flexible suite of utilities for comparing genomic features. *Bioinformatics* **26**, 841–842 (2010).

Ramsey, C. B. & Lee, S. Recent and planned developments of the program OxCal. *Radiocarbon* **55**, 720–730 (2013).

Reimer, P. J. *et al.* The IntCal20 northern hemisphere radiocarbon age calibration curve (0-55 cal kBP). *Radiocarbon* **62**, 725–757 (2020).

Vargas-Ramírez, M., Maran, J. & Fritz, U. Red- and yellow-footed tortoises (*Chelonoidis carbonaria*, *C. denticulata*) in South American savannahs and forests: Do their phylogeographies reflect distinct habitats? *Org. Divers. Evol.* **10**, 161–172 (2010).

Wingett, S. W. & Andrews, S. FastQ Screen: A tool for multi-genome mapping and quality control [version 2; referees: 4 approved]. *F1000Research* **7**, 1338 (2018).
